# Lameness detection in dairy cows from overhead view: high-precision keypoint localization and multi-feature fusion classification

**DOI:** 10.3389/fvets.2025.1675181

**Published:** 2025-09-09

**Authors:** Weijun Duan, Fang Wang, Honghui Li, Na Liu, Xueliang Fu

**Affiliations:** ^1^College of Computer and Information Engineering, Inner Mongolia Agricultural University, Hohhot, China; ^2^Key Laboratory of Agricultural and Pastoral Big Data Research and Application, Hohhot, China; ^3^National Centre of Technology Innovation for Dairy-Breeding and Production Research Subcentre, Hohhot, China; ^4^College of Animal Science, Inner Mongolia Agricultural University, Hohhot, China; ^5^Key Laboratory of Smart Animal Husbandry at Universities of Inner Mongolia Autonomous Region, Integrated Research Platform of Smart Animal Husbandry at Universities of Inner Mongolia, Inner Mongolia Herbivorous Livestock Feed Engineering Technology Research Centre, Hohhot, China

**Keywords:** dairy cows, lameness detection, overhead view, keypoint detection, RGB-D, multi-feature fusion classification

## Abstract

**Introduction:**

Detecting lameness in dairy cows from an overhead view can effectively avoid occlusion caused by farm facilities or other animals, while suspended detection devices enable parallel monitoring without disturbing natural behaviors. However, existing methods from this perspective still face challenges in accuracy and generalization, largely due to the subtlety of back movement features and individual variability. To address these limitations, this study explores an overhead-view lameness detection approach based on RGB-D data.

**Methods:**

We developed a high-precision keypoint detection method for the cow’s back that models long-range spatial dependencies and optimizes structural representation. On this basis, six lameness-related features were designed to capture posture and motion abnormalities, including four newly proposed indices. Their correlation in classifying sound, mildly lame, and severely lame cows was systematically analyzed. To further enhance robustness, the Gini importance index from Random Forest combined with a permutation importance correction method (PIMP) was applied to construct an unbiased feature selection framework.

**Results:**

Experimental results demonstrate that the proposed keypoint detection network achieved a PCK@0.02 of 100.00% and an average precision of 95.89%, significantly outperforming the baseline model. In feature-based classification, back curvature, movement asymmetry index, and vertical oscillations of the back and head exhibited strong discriminative ability. Using multi-feature fusion, the lameness detection model attained an overall accuracy of 91.00%.

**Discussion:**

These findings indicate that overhead RGB-D imaging, combined with precise keypoint detection and feature fusion, provides a reliable strategy for accurate lameness detection in dairy cows. The proposed method offers valuable theoretical and technical support for health monitoring and intelligent management in modern dairy farming.

## 1 Introduction

Lameness is widely recognized as a significant health challenge in the dairy industry. A systematic review encompassing 53 studies across six continents, primarily drawing data from Europe and North America, revealed an average prevalence rate of 22.8% for lameness in dairy cows, with reported prevalence ranging from 5.1 to 45% across these studies (1). Recently, a comprehensive analysis of 38 studies conducted in tropical Asian countries indicated that the average prevalence of lameness in dairy cows in this region is 15.1% (95% confidence interval: 13.0–17.5%) (2). These findings suggest that the incidence of lameness in dairy cows remains high across various regions, presenting a serious challenge to the sustainable development of the industry. In practical production, lameness behavior in dairy cows is frequently grossly underestimated (3), leading to substantial economic losses, which can be categorized into three primary components: milk production losses of about 40%, fertility impairment costs of approximately 30%, and treatment costs also around 30% (4). Given the traditional manual lameness detection methods, which are often highly subjective and time-consuming, there is an urgent need to develop an efficient and automated cow lameness detection system (5, 6). This can facilitate early lameness detection and enable timely manual intervention, thereby reducing economic losses for farms and enhancing the welfare standards of dairy cows.

Lameness in dairy cows leads to significant alterations in gait, typically characterized by an arched back, head nodding, abnormal gait, reduced walking speed, and loss of symmetry (7, 8). Cows affected by lameness usually adopt a series of motion changes to minimize claws or limbs loading and maintain body balance; these abnormal behaviors and postures become increasingly pronounced as the severity of lameness escalates. Based on the features utilized for lameness detection, automated lameness detection methods can be classified into non-kinematic and kinematic categories. Non-kinematic methods focus on the indirect monitoring of physiological and production indicators, such as claws weight-bearing, local body temperature, milk production, and activity levels, to infer the occurrence of lameness. In contrast, Kinematic methods rely on changes in movement patterns caused by lameness, primarily employing motion sensors or computer vision technology to quantitatively analyze and differentiate specific movement characteristics of dairy cows (9–11).

In non-kinematic lameness detection methods, researchers have conducted studies based on claw weight-bearing and thermal imaging detection techniques. For instance, Liu et al. (12) utilized force plates to measure claw ground pressure and discovered that lame dairy cows exhibited significant differences from healthy individuals in terms of peak force and pressure parameter distribution. This finding suggests that weight-bearing distribution can serve as a crucial basis for lameness identification. Similarly, Lin et al. (13) employed infrared thermal imaging technology to monitor claw temperature, revealing that lame cows exhibited significantly higher claw temperatures than their healthy counterparts (*p* < 0.001), with temperature variations showing a strong correlation with clinical lameness scores. However, both methods have inherent limitations. Claw weight-bearing detection generally operates under the assumption that “dairy cows will transfer their weight from the affected limb to the healthy limb.” During the detection process, the individual must remain on the platform for a specified duration to obtain data such as peak force, average vertical force, and limb weight-bearing transfer frequency, which restricts its application in large-scale farms (14). The effectiveness of lameness detection using infrared thermal imaging relies on the premise that “lameness is accompanied by limb inflammation that generates local thermal signals,” but this premise is not universally applicable. Even in the presence of inflammation and fever, factors such as claw dirt coverage, limb moisture levels, and direct sunlight can interfere with the stability of temperature signals, thereby diminishing the reliability of detection (15–17).

Lameness detection based on motion sensors typically involves the attachment of sensors, such as accelerometers, to the limbs or neck of dairy cows to collect motion data. This data is subsequently combined with feature extraction and classification algorithms for the automatic identification of abnormal gait patterns (18). In early studies, Haladjian et al. (19) designed a wearable sensor system that successfully collected hind limb gait information from dairy cows, achieving a lameness detection accuracy of 91.1% under controlled experimental conditions. Research has gradually expanded from single-site signals to the fusion of multi-site signals and multi-source data. For instance, Gertz et al. (20) combined motion data from neck and leg sensors with farm health records, achieving an AUROC of 86% and an F-Measure of 81% in real farming environments. These results indicate that multi-site monitoring and multi-source data fusion possess significant application potential on actual farms. Further research has begun to focus on critical issues such as the early detection and grading of lameness. Thorup et al. (21) analyzed leg movement data from 348 Holstein dairy cows across four commercial farms, discovering that variables such as walking duration and movement indices could effectively distinguish lameness grades at an early stage, providing empirical evidence for the use of movement characteristics in early lameness detection. Although motion sensor-based methods offer certain advantages regarding accuracy, they necessitate the fitting of each cow with a device, which may induce stress in the animals. Additionally, these methods incur high labor and equipment costs and often suffer from poor sensor data stability. The indicators utilized to assess lameness primarily depend on gait or activity features, rendering them vulnerable to interference from other abnormal behaviors (22–25). Conversely, computer vision-based lameness detection has emerged as a prominent research focus due to its non-contact nature, capability to monitor groups, ease of integration with existing farm infrastructure, and excellent scalability. These methods capture walking images of dairy cows using 2D/3D cameras and extract lameness-related features, such as walking speed, stride length, back arch curvature, and key point movement trajectories, thereby facilitating the automatic identification of lameness behavior and the grading of its severity (26–29).

Existing computer vision-based lameness detection methods primarily utilize side posture and motion states in horizontal views for analysis. This approach necessitates the creation of dedicated camera deployment spaces and is vulnerable to obstructions from fences and interference from complex backgrounds. In contrast, lameness detection from an overhead view offers several significant advantages, including a reduced equipment footprint, enhanced resistance to fouling, the absence of occlusion, and the ability to detect multiple targets in parallel. Recent attempts have been made to apply this method for detecting lameness behavior in cows. Studies have demonstrated that effective detection of cow lameness behavior can be achieved using RGB and Depth images from an overhead view. For instance, Zin et al. (30) extracted dorsal spine sequence information from overhead view depth data. They utilized the SVM algorithm to classify lameness based on the average height of the spine, achieving a discriminatory accuracy of over 70% between lame and non-lame cows. Tun et al. (31) reported a maximum detection accuracy of 81.1% by extracting the depth value of the highest point on the cow’s back and employing multiple machine learning classifiers for lameness discrimination. Xin et al. (32) attained an accuracy of 88.7% in lameness detection through feature extraction and classification of spatio-temporal streaming fusion images using an improved PP-TSMv2 network. Zhang et al. (33) achieved lameness detection with an accuracy of 83.05% by identifying keypoints such as the hook bone and tail bone and analyzing their movement trajectories. Collectively, these studies validate the feasibility of lameness detection in dairy cows from an overhead view, while also observing that the accuracy of detection is typically low.

Compared to the horizontal viewpoint method, the lameness movement features of cows observed from an overhead view are relatively inconspicuous, which significantly limits the accuracy of detection. Furthermore, existing studies have shown a limited capacity to adequately express the features of motion changes and abnormal postures, which further impacts the accuracy of lameness detection. Overall, lameness detection in dairy cows from an overhead view predominantly relies on keypoint localization to extract features of motion changes and abnormal postures (34, 35); thus, the precise detection of keypoints is fundamental to enhancing lameness detection accuracy. Additionally, in practical production environments, cows often exhibit complex and varied postural and gait changes due to individual differences in pain locations and motion change strategies (27, 36). A single feature may only reflect localized aspects of abnormal gait, making it prone to overlook or misjudge individuals with atypical features. Some researchers have proposed the use of multi-feature fusion to enhance the effectiveness of lameness detection (35). However, when too many features are included, the presence of invalid or redundant features may introduce noise, thereby diminishing the model’s accuracy and robustness (29). Consequently, accurately detecting the keypoints of a cow’s back from an overhead view, enhancing the feature expression of lameness, and conducting feature screening and multi-feature fusion for lameness classification have emerged as critical issues for a system for detecting cow lameness.

This paper proposes a method for detecting cow lameness based on RGB-D data captured from an overhead view. The main innovations are as follows:

1. A high-precision cow back keypoint detection method based on an overhead view is introduced, establishing a foundation for quantifying motion change and abnormal posture features.
2. Six types of lameness features are designed, four of which are proposed for the first time. The correlation of these features in classifying sound, mild lameness, and severe lameness cows is verified, providing a more accurate representation of the kinematic changes in cow lameness behavior.
3. The Gini importance index of Random Forest is utilized to assess the significance of each feature. To address potential statistical bias, the PIMP correction method is introduced, constructing an unbiased feature screening system that identifies lameness behavior and severity. This method provides both theoretical and practical support for developing an efficient and robust automated system for detecting cow lameness.

## 2 Materials and methods

### 2.1 Criteria for evaluating lameness in dairy cows

The 5-point gait scoring system proposed by Sprecher et al. (37) is among the most widely utilized methods for assessing lameness in dairy cows. However, Zhao et al. (36) discovered that in practical production applications, the distribution of cows corresponding to different scores in the 5-point gait scoring method is highly uneven, with certain scores exhibiting extremely low sample proportions. Directly applying this method would lead to a significant bias in the model toward categories with larger sample sizes. To address the issue of sample imbalance and align the scoring system more closely with the actual needs of clinical interventions on dairy farms, Zhao et al. consolidated the 5-level scoring system into a 3-level system, where scores of 1–2 are classified as sound cows, scores of 3–4 indicate cows with mild lameness, and a score of 5 denotes cows with severe lameness. This classification strategy not only effectively enhances sample distribution and improves the stability of the classification model but also facilitates the direct mapping of classification results to intervention measures in production management, significantly increasing the practical value of the system. Consequently, this study also adopted a 3-level lameness evaluation standard, categorizing dairy cows into sound, mild lameness, and severe lameness, as illustrated in Table 1.

**Table 1:** Criteria for evaluating lameness in dairy cows.

Score	Degree	Standard of judgment
1	sound	The back remains level during standing and walking; the gait appears normal, even if there is slight arching of the back while walking.
2	mild lameness	Arching of the back when standing and walking, with the walking gait affected by short strides in one or more limbs.
3	severe lameness	The back is consistently arched, and each step during walking appears sluggish and deliberate, exhibiting a marked preference or reluctance to bear weight on one or more limbs.

### 2.2 Dataset

#### 2.2.1 Data collection and preprocessing

**Figure 1:**
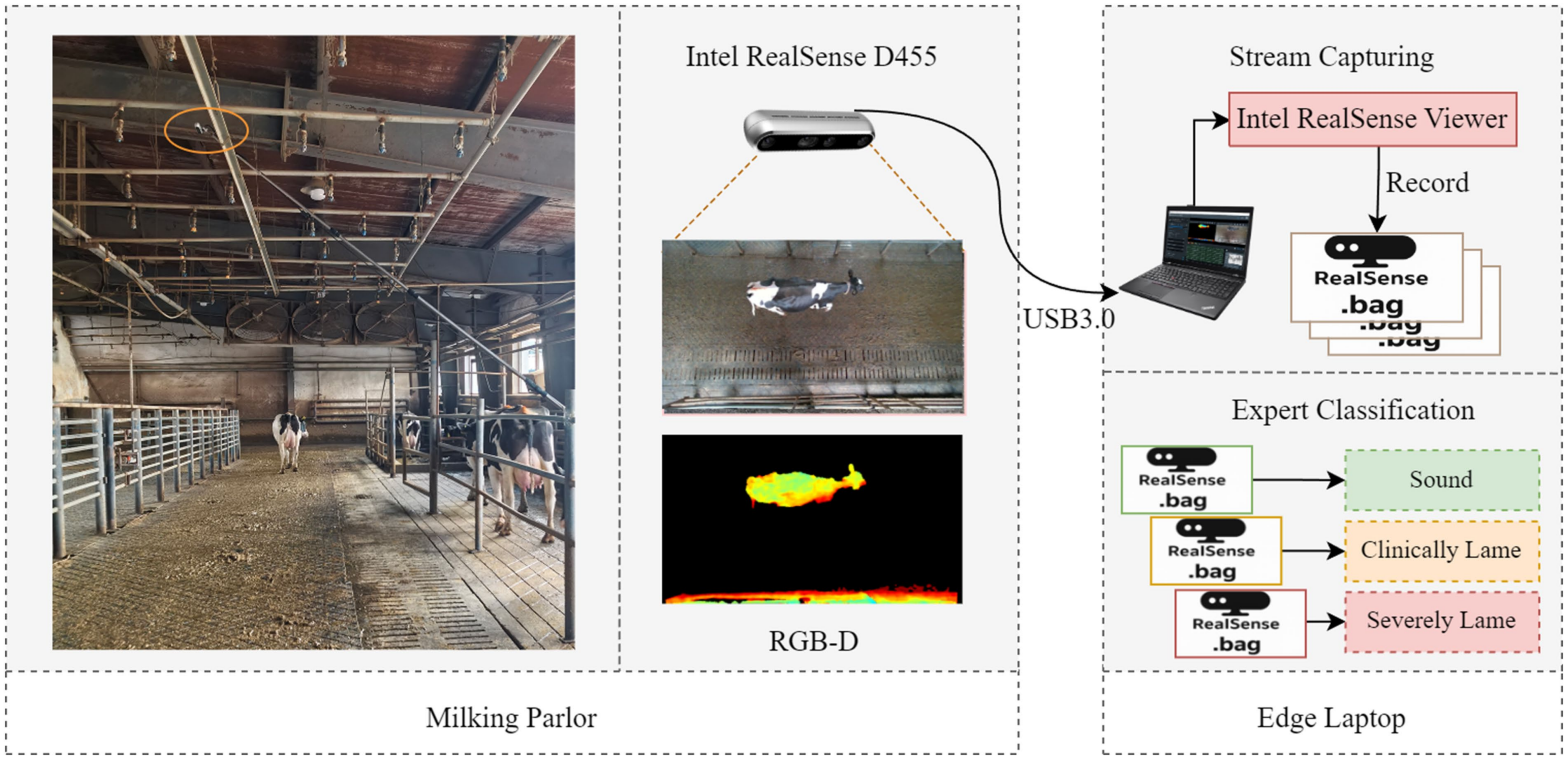
Data acquisition.

In this study, videos of cows walking under natural light conditions were collected between March 7 and April 16, 2025, at a large-scale dairy farm in Hohhot, Inner Mongolia. The farm houses approximately 6,800 Holstein cattle, including over 3,500 lactating cows. The experiment selected the passageway after milking as the data collection area. This passageway measures 28 meters in length and 4.5 meters in width. A RealSense D455 depth camera (manufactured by Intel Corporation, USA) was vertically installed 3.2 meters above the center of the passageway to simultaneously capture RGB and depth video from an overhead view. The video resolution is 1280 × 720, with a frame rate of 30 fps. The experiment was conducted by two livestock experts who manually screened cows that had finished lactation in batches according to the criteria shown in Table 1. A cow was classified into one of three categories: sound, mild lameness, or severe lameness, only when both experts agreed on its lameness assessment. The cows were then guided into the data collection corridor, which allowed only one cow to pass through at a time; the majority of sound dairy cows exited through the right-hand guidance channel, as shown in Figure 1. During data collection, staff maintained a distance from the collection area to allow the cows to move at a natural pace. However, they manually removed any cows that lingered in the collection area for extended periods. A P52s laptop (Lenovo, China) was deployed on-site, utilizing Intel RealSense Viewer (v2.56.3) to record an independent .bag file for each cow, with data categorized and stored according to the severity of lameness.

For the collected RGB-D data, the following methods were employed for preprocessing.

1. Data unpacking: The .bag file was unpacked using the pyrealsense2 library, defining a depth range of 1200mm to 2600mm. RGB frames with a resolution of 1280 × 720 pixels, depth pseudo-color frames, and raw depth matrix data were exported sequentially. Ultimately, each video sequence featuring limping was transformed into sets of RGB image sequences, depth pseudo-color image sequences, and raw depth matrix data sequences.
2. Data screening: Initially, each RGB image sequence was meticulously screened to retain only those frames in which the cow fully entered the field of view and moved at a natural gait. This process excluded abnormal sequences characterized by stuttering, prolonged stationary frames, rapid running, or slipping. Subsequently, the corresponding depth pseudo-color image sequences were scrutinized to eliminate abnormal sequences where the void rate in the cow’s body region was equal to or greater than 10%. Ultimately, a total of 741 cow walking sequences were selected, comprising 260 sound sequences, 237 mild lameness sequences, and 244 severe lameness sequences. From the remaining 538 sequences, 2,520 usable RGB images were chosen for keypoint detection.
3. Depth image preprocessing: Bilateral filtering (spatial domain s = 5, grayscale domain r = 0.1) was applied to the raw depth matrix data from 741 dairy cow sequences to suppress random noise. Subsequently, median filtering with a 3 × 3 window size was employed to eliminate outliers caused by isolated textures. To address depth holes, nearest-neighbor interpolation was utilized, ensuring that depth information in the region of interest (ROI) remained intact. Finally, the preprocessed depth matrix data sequence was converted into a grayscale image sequence.
4. Cow and key point labeling: Labelme (V5.5.0) was utilized to label 2520 images of cow backs. A total of eight keypoints were identified: poll, withers, left scapula, right scapula, lumbar region, left tuber coxae, right tuber coxae and sacral tuber.

#### 2.2.2 Dataset construction

In this study, we constructed the lameness motion dataset and the keypoint dataset separately, as illustrated in Table 2. The lameness motion dataset comprises a sequence of walking images of cows, including both RGB images and their corresponding depth images. The keypoint dataset contains RGB images along with their keypoint annotations. Both datasets are divided into training and validation sets in an 8:2 ratio. The lameness behavior dataset comprises 741 sequences of images depicting lame cows, with the training set and validation set containing 593 and 148 image sequences, respectively. Additionally, the keypoint dataset consists of 2,520 RGB images, with the training set and validation set comprising 2,016 and 504 images, respectively.

**Table 2:** Overview of data sets.

Dataset	Sound (n)	Mild Lameness (n)	Severe Lameness (n)	Training set	Validation set	Total
Lameness motion dataset	260	237	244	593	148	741
Keypoint dataset	-	-	-	2016	504	2520

### 2.3 Lameness detection based on RGB-D from an overhead view

**Figure 2:**
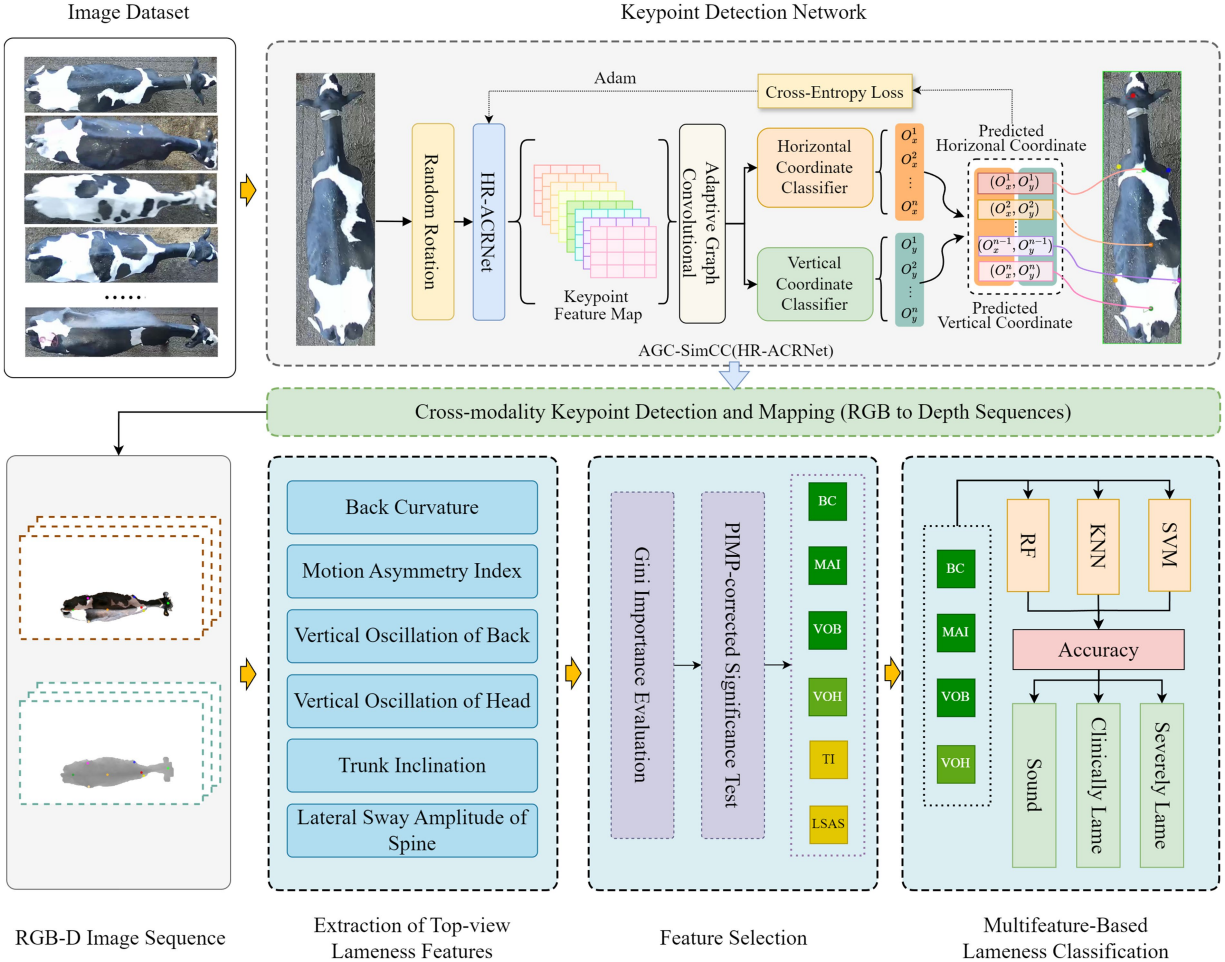
Overall architecture diagram of lameness detection based on overhead view in dairy cows.

This study proposes a method for detecting lameness in dairy cows based on RGB-D images captured from an overhead view. The overall architecture is illustrated in Figure 2 and primarily comprises three components: cow back keypoint detection network, lameness feature construction, feature selection, and multi-feature fusion for lameness classification. First, the keypoint detection network is trained using manually annotated RGB images to ensure accurate localization of keypoints on the backs of dairy cows. Second, by integrating keypoint coordinates with depth image sequences, we extract various overhead features that reflect motion changes and abnormal postures in dairy cows, thereby quantifying different manifestations of lameness. Finally, through statistical analysis and feature selection methods, we assess the correlation of the initial feature set and select the most discriminative feature combinations; Subsequently, we train the optimized feature combination using a multi-feature classifier to facilitate the detection of lameness in dairy cows.

#### 2.3.1 Design of the network for detecting keypoints on cow’s back

Lameness in dairy cows is often accompanied by alterations in back movement patterns and posture. Therefore, accurately reflecting the motion changes and abnormal postures in dairy cows is crucial for effective lameness detection from an overhead view. To further quantify these motion changes and abnormal postures, this study draws upon research related to dairy cow gait and lameness detection (25, 26, 33). Eight key points were selected, including the poll, withers, left scapula, right scapula, lumbar region, left tuber coxae, right tuber coxae, and sacral tuber. These points encompass the central nodes of the spine and reflect the left–right symmetrical structure, thereby enabling the construction of lameness features.

This study employs SimCC (38) as the baseline network for keypoint detection on cow backs. Given the accuracy requirements of SimCC for coordinate regression, HRNet (39) has been selected as the feature extraction backbone. The overall structure is illustrated in the keypoint detection network section of Figure 2. The network comprises four components: data augmentation, backbone feature extraction, Adaptive Graph Convolution, and coordinate classification and regression. Initially, the images are randomly rotated at angles of 0°, 90°, 180°, and 270° to augment the training data and enhance the model’s perception of keypoint directions. Subsequently, HR-ACRNet is constructed for multi-scale feature extraction to mitigate issues such as confusion arising from symmetrical structures. The Adaptive Graph Convolution module is then introduced to explicitly model the spatial dependencies between keypoints, thereby suppressing noise interference from background patterns, dirt, and production marks. Finally, the enhanced features are mapped to the SimCC coordinate classification and regression branches to achieve high-precision keypoint localization.

##### 2.3.1.1 Design of the HR-ACRNet

**Figure 3:**
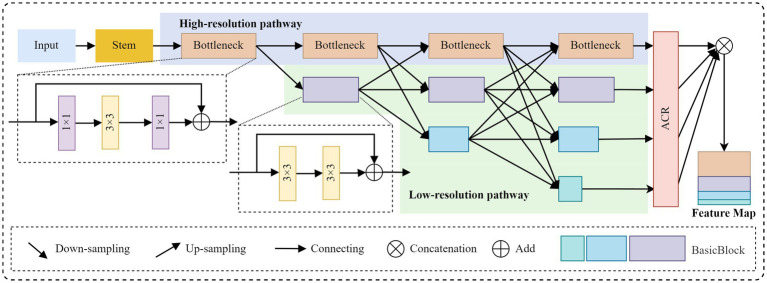
Structure diagram of HR-ACRNet.

**Figure 4:**
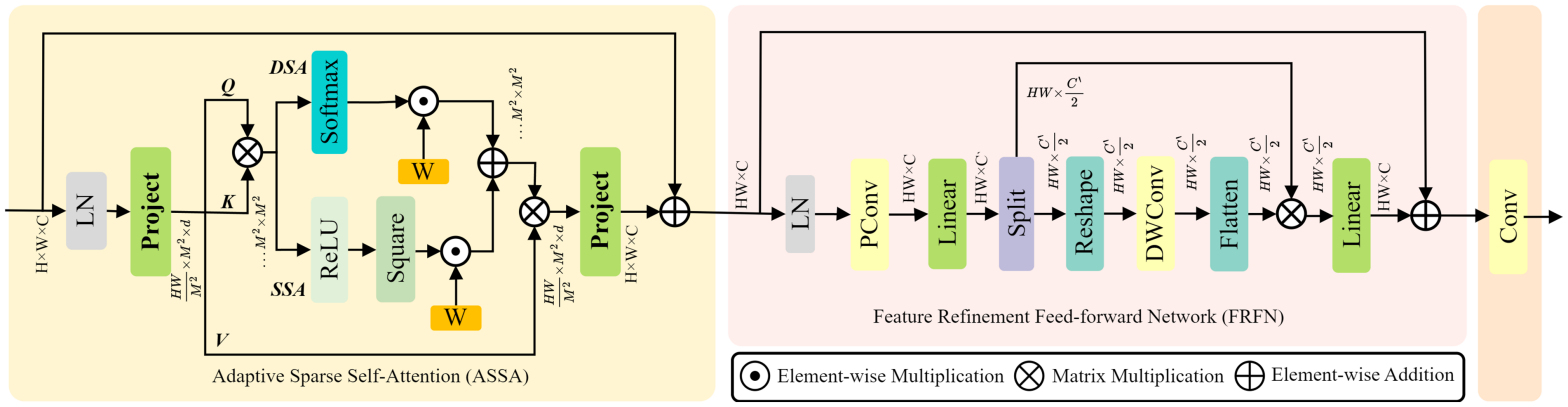
Structure diagram of ACR.

In keypoint detection tasks, HRNet primarily extracts features through local convolution, which limits its ability to capture sufficient global context information. This limitation can lead to confusion between left and right symmetrical keypoints. To address this issue, this study introduces the Adaptive Context Refinement (ACR) module (40), which constructs the HR-ACRNet feature extraction network (as illustrated in Figure 3) to mitigate problems associated with symmetrical structure confusion. The ACR module comprises two components: Adaptive Sparse Self-Attention (ASSA) and Feature Refinement Feedforward Network (FRFN), as depicted in Figure 4. The ACR effectively captures long-range dependencies in both horizontal and vertical directions within the spatial domain through ASSA, while the FRFN integrates global contextual information in the channel domain, thereby significantly enhancing the accuracy of keypoint localization.

Initially, the input image is mapped to $\frac{1}{4}$ feature maps at the base resolution through two layers of 3×3 convolutions with a stride of 2 in HRNet, followed by the extraction of high-resolution features via a series of Bottleneck modules. Subsequently, multiple branches with $\frac{1}{8}$ , $\frac{1}{16}$ , $\frac{1}{32}$ initial resolution are constructed in parallel at each level, facilitating multi-scale information fusion through various upsampling and downsampling processes at each stage. Before HRNet up-samples the four groups of branch features to the highest resolution in equal proportions, an ACR module is introduced on each branch to perform adaptive spatial and channel domain refinement on the output high-resolution features. This module effectively integrates horizontal and vertical long-range dependencies while preserving the high-resolution details of HRNet throughout the process, thereby providing more discriminative features for subsequent keypoint location regression.

In the ASSA layer, the features $X\in {\mathbb{R}}^{H\times W\times C}$ produced by HRNet are partitioned into multiple non-overlapping M×M windows, and the i -th window is flattened to yield $X_{i}\in {\mathbb{R}}^{M^{2}\times C}$ . Employing shared linear transformations to produce Query, Key, and Value matrices, defined as $Q=X_{i}W_{Q},K=X_{i}W_{K},V=X_{i}W_{V}$ , $W_{Q},W_{K},W_{V}\in {\mathbb{R}}^{C_{r}\times d}$ , and d represents the attention dimension for each head. The learnable position bias is $B\in {\mathbb{R}}^{M^{2}\times M^{2}}$ ; hence, the classic dense self-attention (DSA) and sparse self-attention (SSA) are computed by Equations 1, 2, respectively. Then, perform a weighted fusion of DSA and SSA.


(1) $$\mathrm{DSA}=\text{Softmax}(QK^{⊤}/\sqrt{d}+B)$$

(2) $$\mathrm{SSA}={\text{ReLU}}^{2}(QK^{⊤}/\sqrt{d}+B)$$

Among them, Softmax(·) is the normalization operation for rows, ${\text{ReLU}}^{2}(x)=\max{\left(x,0\right)}^{2}$ .

In the FRFN layer, additional computations are conducted on the refined spatial features, adhering to the enhancement-simplification principle, as illustrated in Equation 3. The accuracy of keypoint localization is enhanced by the comprehensive integration of global contextual information in both horizontal and vertical dimensions.


(3) $$\begin{array}{ll} \hat{X} & =\text{GELU}(W_{1}\;\text{PConv}(\tilde{X})),\\ \left[{\hat{X}}_{1},{\hat{X}}_{2}\right] & ={\text{Split}}_{\text{chan}}(\hat{X}),\\ {\hat{X}}_{r} & ={\hat{X}}_{1}⊗F(\text{DWConv}(R({\hat{X}}_{2}))),\\ X_{\text{out}} & =\text{GELU}(W_{2}\;{\hat{X}}_{r}) \end{array}$$

Among them, PConv(·) indicates partial convolution; $W_{1},W_{2}\in {\mathbb{R}}^{C_{r}\times 2C_{r}}$ is channel-by-channel linear mapping; GELU(·) is the gaussian error linear units; ${\text{Split}}_{\text{chan}}(\cdot )$ represents the operation of dividing into two equal parts according to the channel dimension; R(·),F(·) reform and flattening operations that represent mutual transformation between sequences and two-dimensional space; DWConv(·) indicates channel-wise separable convolution; ⊗ represents matrix multiplication between channels; ${\hat{X}}_{1}⊗F(\cdot )$ responsible for simplifying redundant dimensions by channel.

##### 2.3.1.2 Adaptive graph convolutional

**Figure 5:**
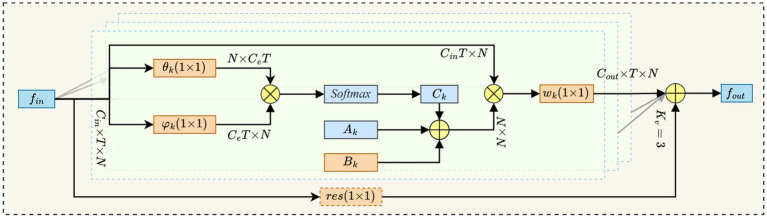
Adaptive graph convolution.

This study employs the SimCC network to detect keypoints on the backs of dairy cows. In comparison to traditional keypoint localization methods that rely on heat maps, SimCC significantly reduces the quantization error associated with pixel grid conversion, thereby enhancing the accuracy of keypoint localization. However, in real-world production environments, individual variations in the patterns on the backs of dairy cows, along with dirt adhesion and production marks, create local high-frequency noise in the images. This noise interferes with the accuracy of SimCC’s coordinate regression, resulting in a shift in the fitted distribution. To mitigate this issue, the study introduces an Adaptive Graph Convolutional (AGC) module (see Figure 5) following the SimCC keypoint feature map (41). By dynamically optimizing the graph’s topological structure and enhancing the spatial interaction of keypoint information, the AGC module effectively suppresses noise interference and improves positioning robustness.

In our study, we denote the input feature map as $X\in {\mathbb{R}}^{C_{\text{in}}\times T\times N}$ and the output feature map as $Y\in {\mathbb{R}}^{C_{\text{out}}\times T\times N}$ , where $C_{\text{in}}$ represents the number of feature channels, T indicates the time length, and N corresponds to the number of keypoints. We employ Equation 4 to execute adaptive graph convolution calculations.


(4) $$Y=\sum_{k=1}^{K_{\upsilon }}W_{k}\;X\;(A_{k}+B_{k}+C_{k})$$

Where, $K_{v}$ denotes the number of spatial subsets, specifically $K_{v}=3$ . $W_{k}\in {\mathbb{R}}^{C_{\text{out}}\times C_{\text{in}}\times 1\times 1}$ represents the 1×1 convolution weight associated with the k -th subset. Additionally, $A_{k},B_{k},C_{k}\in {\mathbb{R}}^{N\times N}$ refer to the predefined adjacency matrix, the learnable compensation matrix, and the data-driven graph, respectively.

$A_{k}$ is obtained by symmetric normalization, as shown in Equation 5.


(5) $$A_{k}=\Lambda_{k}^{-\frac{1}{2}}\;{\bar{A}}_{k}\;\Lambda_{k}^{-\frac{1}{2}}$$

Among them, the elements of ${\bar{A}}_{k}\in {\left\{0,1\right\}}^{N\times N}$ are equal to 1 if and only if the keypoint $v_{j}$ belongs to the k -th neighborhood subset of $v_{i}$ ; $\Lambda_{k}$ is a diagonal normalized matrix.

The elements of the learnable compensation matrix $B_{k}\in {\mathbb{R}}^{N\times N}$ are optimized synchronously with the network during training and are initialized to zero, which enhances the flexibility of the graph structure while preserving the predefined topology.

Data-driven graph $C_{k}$ is calculated based on an embedded Gaussian similarity function, as shown in Equations 6, 7.


(6) $$f(v_{i},v_{j})=\frac{\exp(\theta {\left(v_{i}\right)}^{T}\phi (v_{j}))}{\sum_{J\prime =1}^{N}\exp(\theta {\left(v_{i}\right)}^{T}\phi (v_{j\prime }))}$$

Among them, θ,ϕ are both 1×1 convolutions of the dimension reduction mapping; $\theta (v_{i}),\phi (v_{j})\in {\mathbb{R}}^{C_{e}\times T\times 1}$ are vector representations after the channels are reduced to $C_{e}$ .


(7) $$C_{k}=\text{softmax}(X^{T}\;W_{\theta_{k}}^{T}\;W_{\phi_{k}}\;X)$$

Where, $W_{\theta_{k}},W_{\phi_{k}}\in {\mathbb{R}}^{C_{e}\times C_{\text{in}}\times 1\times 1}$ are the corresponding 1×1 convolution kernels.

Finally, the convolution output Y′ is added to the residual input X , as shown in Equation 8, ensuring the lightweight deployment and stability of the model.


(8) Y=Y′+ℛ(X)

Among them, if $C_{\text{in}}\neq C_{\text{out}}$ , the residual mapping ℛ(·) is completed by a layer of 1×1 convolution for channel alignment.

#### 2.3.2 Construction of lameness feature

Lameness is one of the most prevalent movement disorders observed in dairy cows. To maintain balance, lame cows frequently exhibit alterations in their movement patterns and abnormal postures. As the severity of lameness escalates, these changes and abnormalities become increasingly pronounced.

Jones (42) evaluated the typical abnormal gait characteristics of lame cows using expert surveys focused on gait aspects. The findings indicated that the importance weights of each characteristic in assessing lameness were as follows: general symmetry (24%), tracking (20%), spine curvature (19%), head bobbing (15%), speed (12%), and abduction and adduction (9%).

Although tracking and speed were included in the lameness scoring system proposed by Jones (42), the overhead view of this study revealed that the distance between the contact points of the front and hind limbs was challenging to measure reliably, rendering the tracking metric unmeasurable. Moreover, in actual observations, cows frequently exhibit random pauses during walking, which makes speed an unreliable indicator of ‘walking ease’ and compromises its validity as a quantitative metric. Consequently, this study selected four gait aspects: general symmetry, spine curvature, head bobbing, and abduction and adduction, as core references and integrated them with RGB-D data from an overhead view to construct six quantifiable lameness features: back curvature (BC), movement asymmetry index (MAI), vertical oscillation of the back (VOB), vertical oscillation of the head (VOH), trunk inclination (TI), and lateral sway amplitude of the spine (LSAS). Specifically, General symmetry corresponds to the movement asymmetry index (MAI) and trunk inclination (TI), which reveal differences in left–right weight distribution and movement patterns. Spinal curvature is represented by back curvature (BC) and vertical oscillation of the back (VOB), which characterize the degree of kyphosis and dynamic fluctuations in key regions, respectively. Nodding corresponds to the vertical oscillation of the head (VOH), reflecting the amplitude of head up-and-down movements. Abduction and adduction are indirectly represented by lateral sway amplitude of the spine (LSAS), characterizing lateral gait abnormalities. The aforementioned six features are derived from typical gait characteristics established through expert consensus and form a complementary relationship in terms of quantification, collectively constructing a comprehensive and representative system of gait abnormality features.

**Figure 6:**
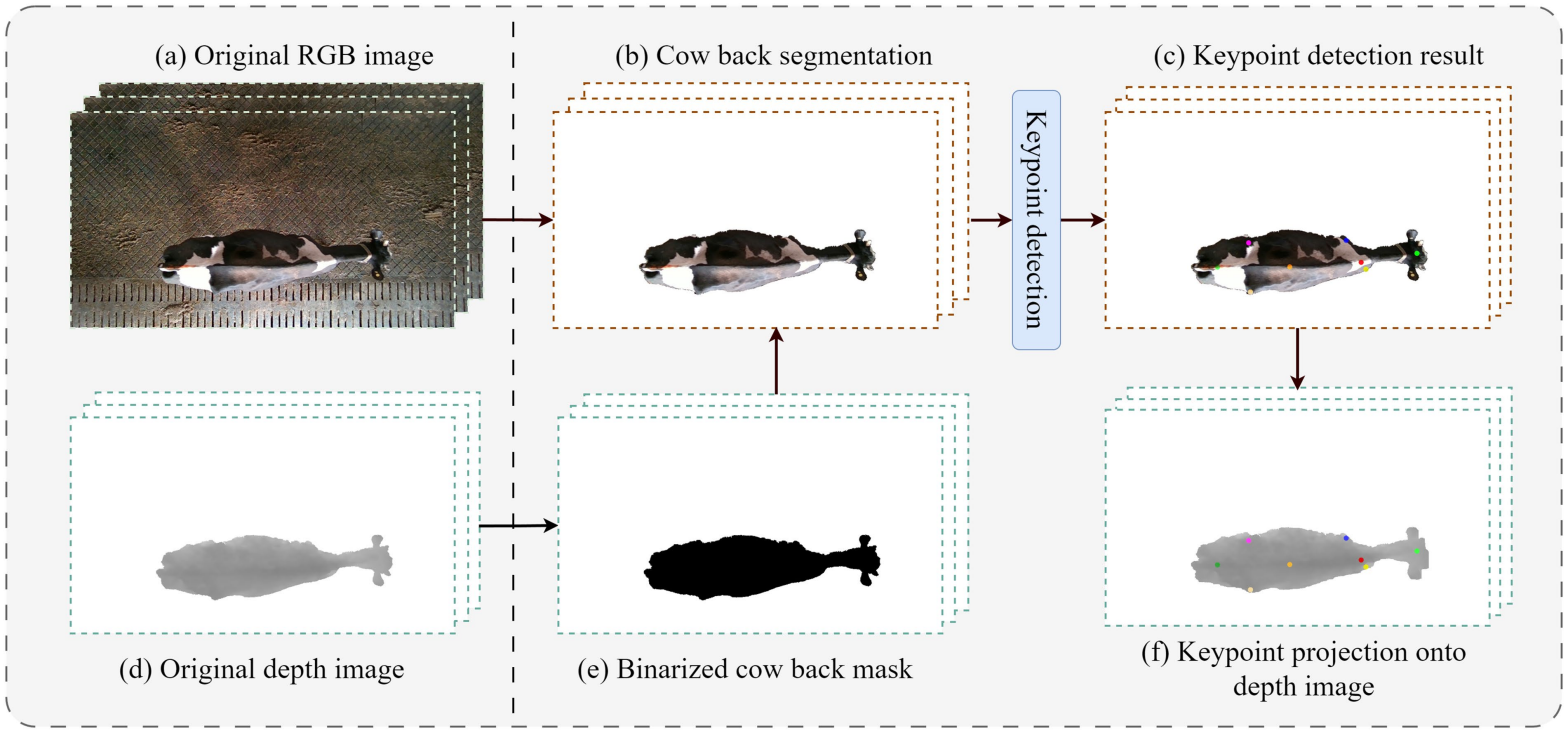
Schematic diagram of RGB-D image preprocessing and keypoint detection process. Panel **(a)** presents the original RGB frame; panel **(b)** displays the cattle segmentation results derived from either semantic segmentation or threshold extraction; panel **(c)** shows the detection of eight keypoints located on the back within the RGB image. Panel **(d)** depicts the original depth frame; panel **(e)** presents the cow body area obtained by applying a binarization mask to the depth map; and panel **(f)** indicates that the keypoint coordinates extracted from panel **(c)** are mapped back to the depth map to extract corresponding depth values.

As illustrated in Figure 6, following preprocessing, all image sequences yield RGB image sequences that include keypoint coordinates and corresponding depth image sequences. Building upon this, and in conjunction with the keypoint annotation results, six overhead view limping features are computed. The specific calculation method is detailed below.

1. Back curvature: It is utilized to quantify the degree of spinal curvature. In the overhead view, the positions of the withers, lumbar region, and sacral tuber are designated as $p_{1}$ , $p_{2}$ , $p_{3}$ , respectively. A circle with radius R is fitted around these points. The back curvature, denoted as $k=\frac{1}{R}$ . The value of k is calculated frame by frame, with the maximum value taken as the representative feature of back curvature.
2. Movement asymmetry index: It is utilized to quantify the disparity in motion intensity between the left and right sides of a cow’s back. Initially, the foreground mask of the dairy cows is extracted based on the depth map. The back is then divided into left and right halves using the normal direction of the line connecting the withers ( $p_{1}$ ) and the lumbar region ( $p_{2}$ ). Subsequently, Farneback optical flow is applied to each RGB image frame to compute the dense velocity field $F(x,y)={\left[u(x,y),v(x,y)\right]}^{T}$ . Following this, the light flow amplitude field is defined as M(x,y)=∥F(x,y)∥ , and the mean light flow amplitudes $\mu_{L}$ and $\mu_{R}$ in the left and right halves ( $\Omega_{L}$ and $\Omega_{R}$ , respectively) are calculated. Finally, the asymmetry index IMAI is computed using Equation 9, which reflects the difference in motion intensity between the two sides.

(9) $$I_{\mathrm{MAI}}=\frac{|\mu_{L}-\mu_{R}|}{\mu_{L}+\mu_{R}}$$

1. Vertical oscillation of the back: This metric quantifies the dairy cows’ vertical oscillation of the back during walking. Calculate the difference between the maximum and minimum values of the temporal depth at the withers, lumbar region, and sacral tuber. Subsequently, determine the maximum value among these three differences to represent the vertical oscillation of the back.
2. Vertical oscillation of the head: This metric quantifies the vertical oscillation of the head of dairy cows while walking. The vertical oscillation of the head is determined by calculating the difference between the maximum and minimum values of the temporal depth of the poll keypoint in the depth map across the sequence frame.
3. Trunk inclination: This metric quantifies the lateral tilt compensation of dairy cows due to uneven forces acting on the front or hind limbs. The depth difference between the left and right scapulae and between the left and right tuber coxae is calculated in each frame, and the maximum absolute value of these left–right depth differences is used to quantify trunk inclination.
4. Lateral sway amplitude of the spine: This metric quantifies the lateral sway amplitude of the spine of dairy cows during walking. The peak-to-valley difference is calculated between the center of the shoulder blades, the lumbar region, and the sacral tuber in the horizontal direction of the image, with the maximum value among the three representing the lateral sway amplitude of the spine.

#### 2.3.3 Feature selection and multi-feature fusion for lameness classification

This study employs Random Forest (RF) to assess the importance of six lameness features in distinguishing the severity of lameness. Compared to common feature selection and dimensionality reduction methods, such as Principal Component Analysis (PCA), LASSO, and Recursive Feature Elimination (RFE), RF can effectively model complex nonlinear relationships between features and achieve robust and interpretable feature selection through Gini importance. This makes RF particularly suitable for analyzing multivariate and high-noise data. Considering that feature importance evaluation in tree models may be biased (43), we further introduced the Permutation Importance Correction Method (PIMP) (44), which enhances the objectivity and biological interpretability of feature screening through label permutation and statistical correction.

The specific process of feature screening and multi-feature fusion for lameness classification in this study is outlined as follows: First, we train a Random Forest model utilizing the original labels to calculate the Gini importance of the six features. Next, perform 50 random permutations of the sample labels, retrain the model, and construct the importance distribution of each feature under the null hypothesis. Subsequently, we select the quantile probability of the original feature importance values that are situated to the right of the permutation distribution to derive the *p*-value for the PIMP. And then apply the Benjamini-Hochberg method for multiple hypothesis correction, retaining only those features with a corrected p-value of less than 0.05. Finally, we standardize the selected features to have a mean of zero and a unit variance, concatenate them into the final feature vector, and input them into Random Forest, K-Nearest Neighbors, and Support Vector Machines to systematically evaluate the performance of each model in identifying lameness using the same feature set.

### 2.4 Evaluation indicators

1. This study employs PCK@0.05, PCK@0.02, AP, and AR indicators to assess the performance of keypoint detection networks.

Under the heatmap normalization scale, a detection point is deemed correctly located if the Euclidean distance to the ground truth point is less than the threshold of 0.05 (or 0.02). The PCK is computed by taking the ratio of the number of correctly located keypoints to the total number of keypoints, as illustrated in Equation 10.


(10) $$\mathrm{pck}@0.05=\frac{1}{N}\sum_{i=1}^{N}1(\|p_{i}-g_{i}\|2\leq 0.05.\max(H,W))$$

Among them, $p_{i}$ and $g_{i}$ are the predicted and ground truth coordinates of the i -th keypoint, H×W is the size of the heat map, and 1(·) is the indicator function.

Average Precision measures the proportion of true positive samples that the model detects. In contrast, Average Recall assesses the proportion of all true positive samples that are correctly identified, as demonstrated in Equations 11, 12.


(11) $$\mathrm{AP}=\frac{1}{n}\sum_{i=1}^{n}\frac{{\mathrm{TP}}_{i}}{{\mathrm{TP}}_{i}+{\mathrm{FP}}_{i}}$$

(12) $$\mathrm{AR}=\frac{1}{n}\sum_{i=1}^{n}\frac{{\mathrm{TP}}_{i}}{{\mathrm{TP}}_{i}+{\mathrm{FN}}_{i}}$$

Among them, ${\mathrm{TP}}_{i}$ , ${\mathrm{FP}}_{i}$ , and ${\mathrm{FN}}_{i}$ represent the true positives, false positives, and false negatives at the i -th threshold, respectively, and n denotes the number of thresholds. AP and AR are the average precision and recall values across all thresholds, respectively.

1. This paper employs accuracy, sensitivity, specificity, and Macro $F_{1}$ -score to assess the performance of lameness detection.

Accuracy (ACC) represents the proportion of correctly classified samples among all samples, as defined in Equation 13.


(13) $$\text{Accuracy}=\frac{1}{N}\sum_{i=1}^{N}1(y_{i}={\hat{y}}_{i})$$

Among them, N is the total number of samples, $y_{i}$ is the true label of the i -th sample, and ${\hat{y}}_{i}$ is the predicted label.

Sensitivity (SENS) refers to the proportion of true positives among all actual samples of a specific class, thereby evaluating the model’s ability to identify samples of that class accurately. For class c , the calculation method is detailed in Equation 14.


(14) $${\text{SENS}}_{c}=\frac{{\mathrm{TP}}_{c}}{{\mathrm{TP}}_{c}+{\mathrm{FN}}_{c}}$$

Where ${\mathrm{TP}}_{c}$ is the number of true examples in class c , and ${\mathrm{FN}}_{c}$ is the number of false negatives in class c .

Specificity (SPEC) denotes the proportion of true negative samples relative to the total number of samples in the class, reflecting the model’s capacity to exclude non-class samples. For the c -th class, the calculation method is specified in Equation 15.


(15) $${\text{SPEC}}_{c}=\frac{{\mathrm{TN}}_{c}}{{\mathrm{TN}}_{c}+{\mathrm{FP}}_{c}}$$

Among them, ${\mathrm{TN}}_{c}$ is the number of true negative examples in class c , and ${\mathrm{FP}}_{c}$ is the number of false positive examples in class c .

Macro $F_{1}$ -score is calculated by determining the $F_{1}$ score for each category, as shown in Equation 16, and subsequently computing the arithmetic mean of all categories’ $F_{1}$ -scores to mitigate the effects of class imbalance, as described in Equation 17.


(16) $$F1_{c}=\frac{2\cdot {\text{Precision}}_{c}\cdot {\text{Recall}}_{c}}{{\text{Precision}}_{c}+{\text{Recall}}_{c}}$$

(17) $$\text{Macro}\;F1=\frac{1}{C}\sum_{c=1}^{C}F1_{c}$$

Among them, C represents the total number of categories, and in this study, C=3 .

### 2.5 Experimental environment and parameter settings

This experiment was conducted on an Ubuntu Server 22.04 for model training and evaluation. The hardware configuration comprises two Intel® Xeon® Gold 6139M CPUs (clock speed: 2.30 GHz), 128 GB of RAM, and eight NVIDIA GeForce RTX 3090 graphics cards. The software environment integrates Python 3.10.11, CUDA 11.7, PyTorch 2.0.1, and MMPose 1.3.2, among other deep learning frameworks. Detailed experimental parameters are presented in Table 3.

**Table 3:** Experimental parameter settings.

Hyperparameter	Value
Input resolution	384×192
Batch size	192
Training epochs	300
Optimizer	Adam
Base learning rate	0.001
LR schedule	MultiStep (170, 200 epoch)
SimCC σ	6.0
SimCC split ratio	2.0
Evaluation metrics	COCO AP / AR

## 3 Results

### 3.1 Performance analysis of keypoint detection network and ablation experiment

This study employs SimCC (HRNet) as the baseline and assesses the effectiveness of the enhanced keypoint detection model through a series of ablation experiments. The experiments evaluate the keypoint detection performance of each model using the AP, AR, loss, and PCK@0.02 metrics.

**Figure 7:**
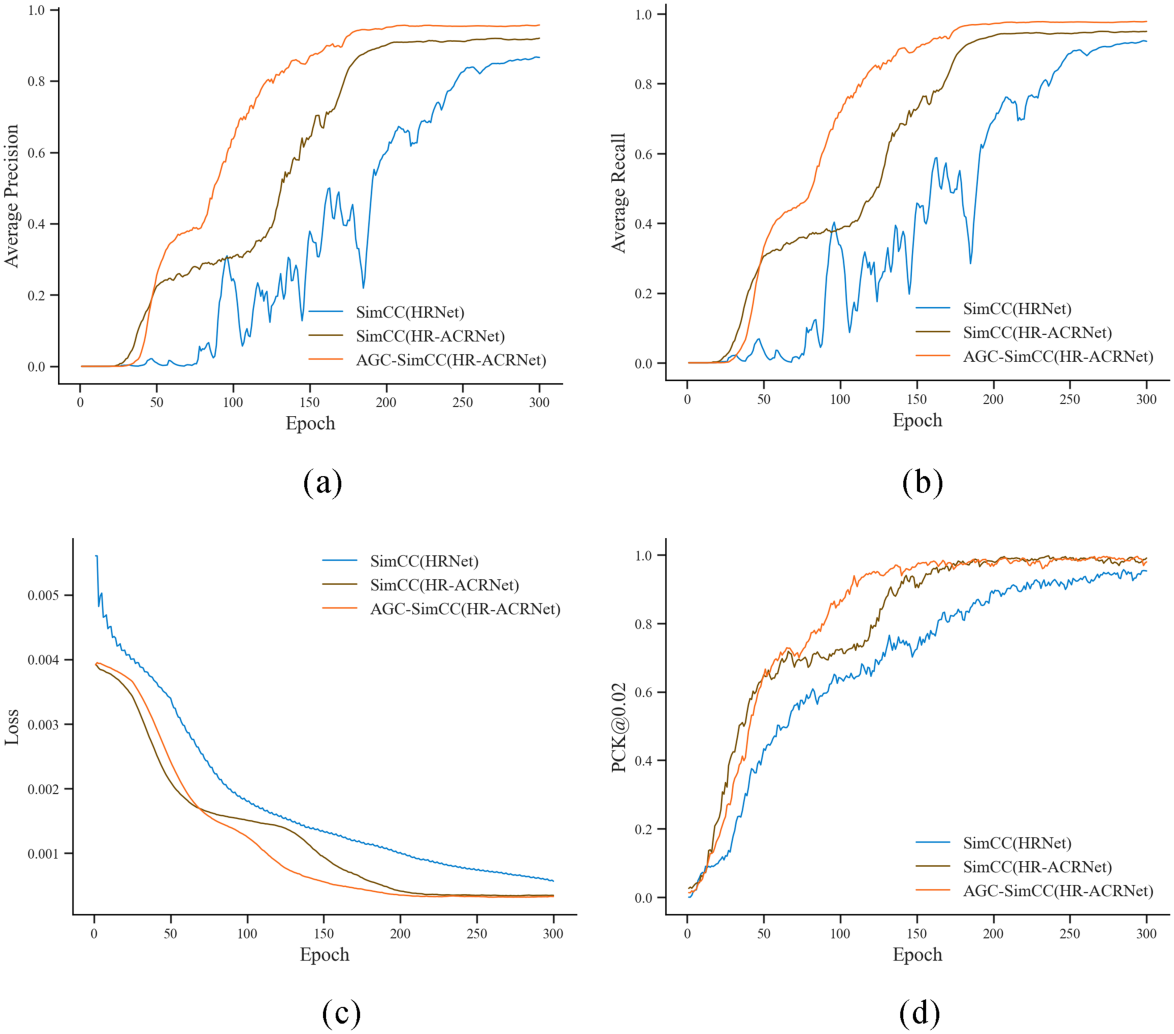
Iterative change curve of key indicators during keypoint detection model training. **(a)** Average precision; **(b)** Average recall; **(c)** Loss; **(d)** PCK@0.02.

Analysis of Figure 7 indicates that, as training iterations progress, the proposed AGC-SimCC(HR-ACRNet) achieves significantly faster convergence rates and superior final performance on PCK@0.02, AP, and AR compared to SimCC(HRNet) and SimCC(HR-ACRNet). Specifically, PCK@0.02 approaches approximately one by the 130th epoch, while AP and AR stabilize around the 170th epoch. The training curves for all three metrics exhibit the fastest initial ascent and the smallest fluctuations. The training loss of AGC-SimCC(HR-ACRNet) converges to below 0.0008 by approximately the 120th epoch. This trend demonstrates that the long-range dependency modeling introduced by the ACR module and the keypoint space structure optimization of the AGC module collaboratively enhance the accuracy and robustness of keypoint detection on the cow’s back, thereby validating the roles of these two modules in mitigating symmetric ambiguity and high-frequency noise.

As shown in Table 4, AGC-SimCC (HR-ACRNet) improved the AP and AR by 8.60 and 5.24 percentage points, respectively, compared to SimCC (HRNet). Under the more stringent PCK@0.02 metric, AGC-SimCC (HR-ACRNet) achieved a keypoint detection rate of 100.00%. This further validates that the improvement strategy proposed in this paper significantly enhances the keypoint detection performance of the model.

**Table 4:** Ablation experiment.

Model	AP (%)	AR (%)	PCK@0.05 (%)	PCK@0.02 (%)
SimCC (HRNet)	87.29	92.70	99.21	98.74
SimCC (HR-ACRNet)	92.33	95.14	100.00	99.36
AGC-SimCC (HR-ACRNet)	95.89	97.94	100.00	100.00

### 3.2 Analyzing the correlation of lameness features

**Figure 8:**
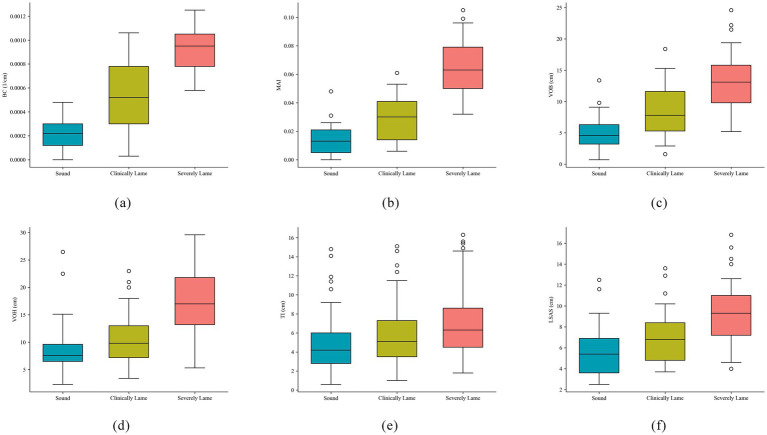
Box plots of six types of lameness features. **(a)** Back curvature; **(b)** Movement asymmetry index; **(c)** Vertical oscillation of back; **(d)** Vertical oscillation of head; **(e)** Trunk tilt asymmetry index; **(f)** Lateral sway amplitude of spine.

Figure 8 illustrates the distribution differences among six lameness features across three groups of lameness samples. In panels (a), (b), and (d), the features BC, MAI, and VOH exhibit overlap between the first two levels, yet they distinctly differentiate severe lameness. Additionally, panel (c) demonstrates a significant capability for lameness discrimination. Conversely, in panels (e) and (f), the features TI and LSAS show considerable overlap across all levels, which complicates the discrimination of lameness.

As demonstrated in Table 5, the mean values of the six lameness features exhibit a consistent trend corresponding to the parameter values of these features. This observation indicates a close relationship between the lameness features and the movement patterns of dairy cows experiencing lameness. However, when considering Figure 8, it becomes evident that the degree of lameness exhibits a nonlinear relationship with movement features. For instance, individuals with mild lameness may display milder back arch abnormalities compared to those with severe lameness, due to individual variability. This phenomenon, while obscured by the average effects in group statistics, can lead to inaccuracies in individual lameness classification. Consequently, it suggests that the feature scoring system based on a linear monotonic assumption does not accurately reflect the underlying pathological processes. Therefore, this study plans to incorporate nonlinear and multifaceted feature threshold classification methods to address these challenges as effectively as possible.

**Table 5:** Estimated mean values of the six lameness indicators.

Degree	BC (1/cm)	MAI	VOB (cm)	VOH (cm)	TI (cm)	LSAS (cm)
Sound	0.00021	0.01200	4.72	7.92	3.63	5.33
Mild lameness	0.00054	0.02700	8.23	10.36	5.18	6.67
Severe lameness	0.00093	0.06400	12.90	17.28	6.76	9.18

### 3.3 Evaluation of feature selection and multi-feature fusion classification

This study conducts evaluations of statistical significance and feature importance for six lameness features, with the results presented in Table 6. Among these features, the intergroup differences for BC, MAI, and VOB reach statistical significance (*p* < 0.001), and the PIMP-corrected *p*-value is also significantly below 0.05, indicating that these three features possess the strongest discriminative ability for lameness classification. The intergroup difference for VOH yields a p-value of 0.0002, while the PIMP-corrected p-value is 0.091. Despite its slightly lower discriminative ability compared to the first three features, VOH still demonstrates a certain level of discriminative capability. Although TI exhibits a high Gini importance (0.19), the PIMP correction does not pass the significance test (*p* = 0.314), suggesting that the importance of this feature lacks statistical support, and all indicators of LSAS are found to be unsatisfactory.

**Table 6:** Statistical significance and feature importance evaluation of six lameness indicators.

Features	Inter-group significance (*p*)	Gini importance	PIMP-corrected *p*-value	Discriminative features
BC	0.0003	0.24	0.014	Yes
MAI	0.0006	0.26	0.010	Yes
VOB	0.0001	0.21	0.022	Yes
VOH	0.0002	0.15	0.091	Weak
TI	0.0011	0.19	0.314	No
LSAS	0.0048	0.06	0.242	No

As shown in Table 7, the optimal feature combination identified in this study is BC + MAI + VOB + VOH, which achieves a classification accuracy of 0.91 and a Macro F_1_-score of 0.85 under the Random Forest model, significantly outperforming both individual features and other feature combinations. Specifically, the feature combination of BC, MAI, and VOB markedly enhances classification performance. The inclusion of boundary features such as VOH further boosts performance; however, the introduction of non-discriminative features like TI and LSAS leads to a decline in classification efficacy. Taking TI as an example, although its intergroup differences are statistically significant (*p* = 0.0011), the p-value after PIMP correction is 0.314, indicating inadequate discriminative power. Experiments also demonstrate that incorporating this feature diminishes the model’s classification accuracy. This suggests that in multi-feature fusion classification, an abundance of features does not necessarily yield better results; rather, judicious feature selection is vital for optimizing the model’s generalization ability and performance.

**Table 7:** Performance evaluation of lameness classification using multi-feature fusion.

Features	Algorithm	ACC	Sound		Mild Lameness		Severe Lameness		Macro $F_{1}$
			SENS	SPEC	SENS	SPEC	SENS	SPEC	
BC	RF	0.77	0.82	0.81	0.61	0.86	0.66	0.93	0.73
	KNN	0.74	0.80	0.80	0.60	0.85	0.64	0.92	0.71
	SVM	0.73	0.80	0.78	0.58	0.84	0.61	0.91	0.70
MAI	RF	0.78	0.84	0.81	0.62	0.87	0.67	0.93	0.74
	KNN	0.77	0.82	0.80	0.61	0.87	0.66	0.92	0.73
	SVM	0.77	0.81	0.79	0.60	0.84	0.65	0.91	0.72
VOB	RF	0.73	0.80	0.79	0.53	0.83	0.61	0.89	0.69
	KNN	0.71	0.78	0.78	0.52	0.81	0.60	0.88	0.67
	SVM	0.70	0.77	0.76	0.51	0.80	0.59	0.87	0.67
VOH	RF	0.71	0.78	0.76	0.50	0.81	0.60	0.88	0.67
	KNN	0.70	0.76	0.76	0.49	0.80	0.59	0.88	0.66
	SVM	0.69	0.75	0.75	0.49	0.80	0.58	0.86	0.65
TI	RF	0.63	0.69	0.72	0.36	0.75	0.44	0.85	0.59
	KNN	0.61	0.67	0.71	0.35	0.74	0.42	0.84	0.58
	SVM	0.62	0.66	0.70	0.35	0.74	0.41	0.83	0.5
LSAS	RF	0.62	0.72	0.74	0.38	0.77	0.45	0.86	0.61
	KNN	0.58	0.70	0.73	0.36	0.75	0.43	0.84	0.59
	SVM	0.59	0.71	0.73	0.35	0.74	0.42	0.83	0.59
BC + MAI + VOB	RF	0.88	0.94	0.94	0.77	0.98	0.80	0.96	0.83
	KNN	0.86	0.92	0.94	0.77	0.96	0.76	0.97	0.81
	SVM	0.82	0.88	0.90	0.72	0.90	0.74	0.91	0.75
BC + MAI + VOB + VOH	RF	0.91	0.96	0.95	0.83	0.97	0.82	0.98	0.85
	KNN	0.88	0.93	0.95	0.88	0.97	0.80	0.97	0.85
	SVM	0.85	0.91	0.92	0.84	0.93	0.79	0.94	0.78
BC + MAI + VOB + VOH + TI	RF	0.86	0.93	0.94	0.77	0.94	0.79	0.96	0.81
	KNN	0.82	0.88	0.90	0.73	0.90	0.75	0.91	0.76
	SVM	0.82	0.87	0.89	0.74	0.91	0.73	0.91	0.75
All features	RF	0.83	0.91	0.92	0.74	0.92	0.76	0.93	0.78
	KNN	0.81	0.87	0.89	0.72	0.89	0.73	0.90	0.75
	SVM	0.79	0.86	0.87	0.69	0.87	0.71	0.88	0.73

## 4 Discussion

This study investigates the alterations in movement and abnormal postures resulting from lameness. It examines three key aspects: the detection of key points on the backs of cows, the construction of lameness features, and the selection and fusion of these features, aiming to effectively differentiate between varying degrees of lameness.

In terms of keypoint detection performance, this study addresses critical factors such as the high symmetry of keypoints on the backs of cows and the interference of high-frequency noise in production scenarios. It proposes the AGC-SimCC (HR-ACRNet) detection method for the first time. Experimental results, as shown in Table 4 and Figure 7, indicate that AGC-SimCC (HR-ACRNet) significantly outperforms the baseline network in terms of PCK, AP, and AR metrics. Specifically, the ACR module enhances long-range dependency modeling and global information integration capabilities through adaptive sparse attention and feature refinement mechanisms. Meanwhile, the AGC module improves the model’s robustness to high-frequency noise by optimizing the spatial topological relationships between keypoints. This design approach aligns with the conclusions drawn by Zhou et al. (40) regarding the superiority of sparse attention in global information modeling. It supports Shi et al. (41) proposal that adaptive graph convolutions enhance structural modeling capabilities.

**Figure 9:**
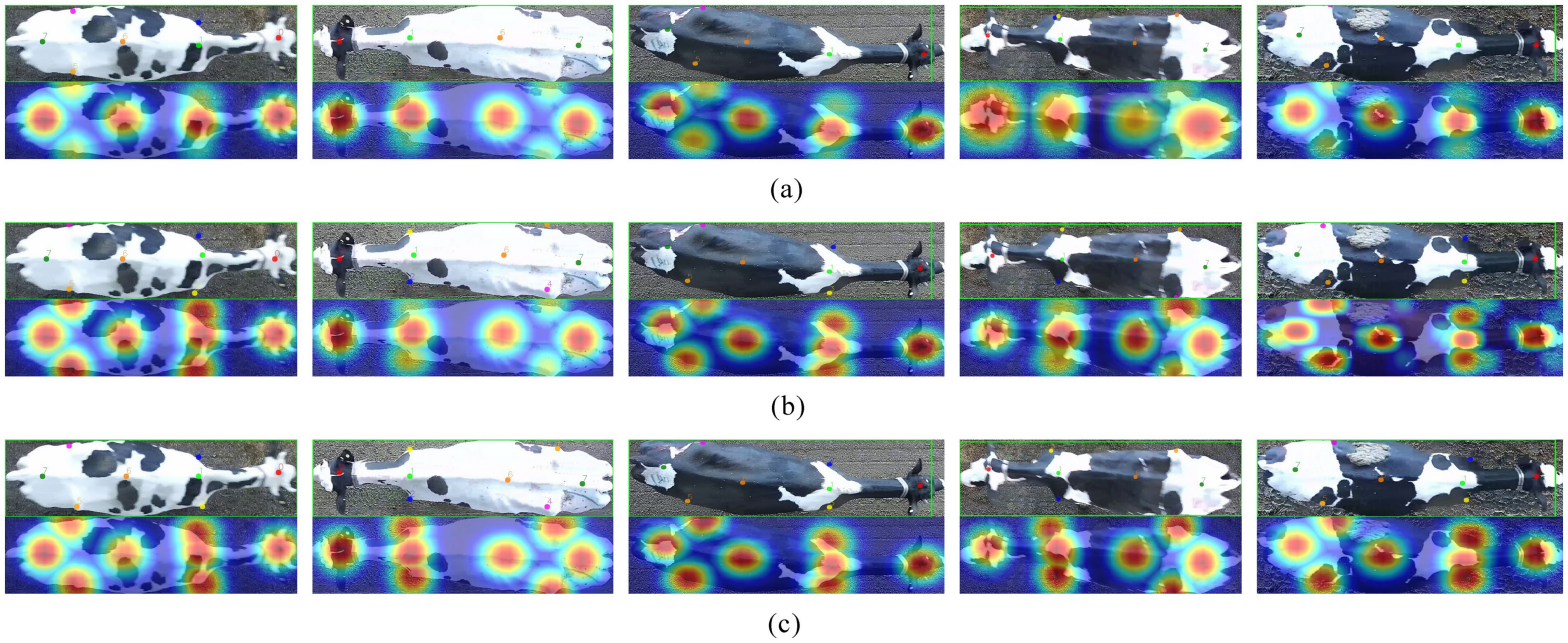
Comparison of keypoint detection results and heat maps of different models. **(a)** SimCC (HRNet); **(b)** SimCC (HR-ACRNet); **(c)** AGC-SimCC (HR-ACRNet).

Figure 9 illustrates the keypoint detection results from the three networks alongside their corresponding CAM heatmaps. In comparison to the baseline network depicted in Figure 9a, the ACR module presented in Figure 9b effectively mitigates the keypoint detection confusion caused by the symmetric structure of the backbone by modeling long-range dependencies. Furthermore, as shown in Figure 9c, the AGC module significantly enhances the accuracy of keypoint localization through the optimization of the spatial topological structure of keypoints. A comprehensive analysis of the CAM heatmaps indicates that the AGC-SimCC (HR-ACRNet) model not only achieves exceptional performance in keypoint localization but also demonstrates greater robustness in mitigating local high-frequency noise interference, such as patterns and dirt. These findings substantiate the model’s efficacy in addressing challenges related to strong symmetry and noise interference in practical applications, thereby establishing a solid foundation for subsequent lameness feature construction.

AGC-SimCC (HR-ACRNet) was compared with prominent keypoint detection networks, including DeepLabCut (45) and LEAP (46), as illustrated in Table 8. The results indicate that AGC-SimCC (HR-ACRNet) exhibits significantly superior detection performance compared to both DeepLabCut and LEAP, particularly under complex conditions characterized by high symmetry of keypoints on the cow’s back and substantial noise. These findings further substantiate that the integration of long-range dependency modeling and graph structure optimization effectively enhances the accuracy of keypoint detection on the backs of cows. The conclusions drawn from this study align with the research conducted by Chen et al. and Zhang et al. (47, 48), which emphasizes the enhancement of robustness in animal pose estimation through graph structures, thereby validating the essential role of higher-order spatial constraints in improving keypoint detection performance.

**Table 8:** Performance comparison of mainstream keypoint detection networks.

Model	AP (%)	AR (%)	PCK@0.05 (%)	PCK@0.02 (%)
AGC-SimCC (HR-ACRNet)	95.89	97.94	100.00	100.00
DeepLabCut (ResNet-101)	88.74	91.20	97.53	95.82
LEAP (FCN)	84.28	89.35	96.02	93.65

From the perspective of feature construction, this study is based on the typical abnormal gait manifestations of lame cows as proposed by Jones (42). It combines these manifestations with research conducted from an overhead view to design six quantifiable lameness features. As illustrated in Figure 8 and Table 6, back curvature (BC), movement asymmetry index (MAI), and vertical oscillation of the back (VOB) demonstrate the most significant discriminative performance in lameness grading. Additionally, vertical oscillation of the head (VOH) offers some discriminative advantage, while trunk inclination (TI) and lateral sway amplitude of the spine (LSAS) contribute relatively less. These findings are consistent with previous studies that highlight the significance of spinal curvature (26, 29), head bobbing (49), and general symmetry (23) in the characterization of limping. Moreover, this research extends these concepts to quantifiable metrics under overhead conditions, thereby providing new dimensions for automated limping detection and enriching the existing system of limping discrimination features.

Spinal curvature is recognized as one of the most apparent external indicators of lameness (26, 29). Back curvature (BC) serves as an effective metric for quantifying the degree of kyphosis, thereby reflecting the abnormal posture of the back associated with lameness. Hind limb claw diseases frequently lead to compensatory movements, such as head-up or hip-up postures, in dairy cows (50). The vertical oscillation of the back (VOB) quantifies this compensatory behavior by assessing the dynamic fluctuations of key points. Asymmetry in movement is a significant characteristic of lameness (23), and the movement asymmetry index (MAI) directly reflects alterations in gait symmetry by characterizing the differences in movement intensity between the left and right sides of the cow’s back. Nodding movements are particularly pronounced when abnormalities are present in the front limbs (49, 50). The vertical oscillation of the head (VOH) quantifies the amplitude of head movement; however, its significance may be constrained due to the proportion of individuals with front limb lameness. Additionally, lame dairy cows often display uneven body weight distribution, which was quantified using trunk inclination (TI) in the experiment. Nonetheless, this indicator relies on the depth values of key points along the edge of the cow’s back, and its accuracy is susceptible to image quality and the precision of key point localization, resulting in limited applicability under the conditions of this study. Abduction and adduction characteristics have been confirmed to correlate with lameness (42); the lateral sway amplitude of the spine (LSAS), as a dorsal mapping of these characteristics from an overhead view, can indirectly reflect lateral gait abnormalities. However, due to the small amplitude of lateral sway, the signal-to-noise ratio is low under current resolution and noise conditions, leading to inadequate quantification effectiveness.

From the perspective of feature selection and fusion, dairy cows exhibit diverse gait changes due to variations in the location of lameness-related pain and individual movement adaptation strategies. Single features often reflect localized manifestations of abnormal movement, which may lead to potential omissions or misjudgments. Consequently, feature fusion has emerged as a significant trend in lameness classification (27, 29). Multi-feature fusion integrates information from multiple kinematic dimensions, thereby enabling effective differentiation of lame individuals. The experimental results of this study (see Table 6) further validate the substantial role of multi-feature methods in improving the limping detection rate and classification sensitivity, consistent with the findings of Russello et al. (35).

**Figure 10:**
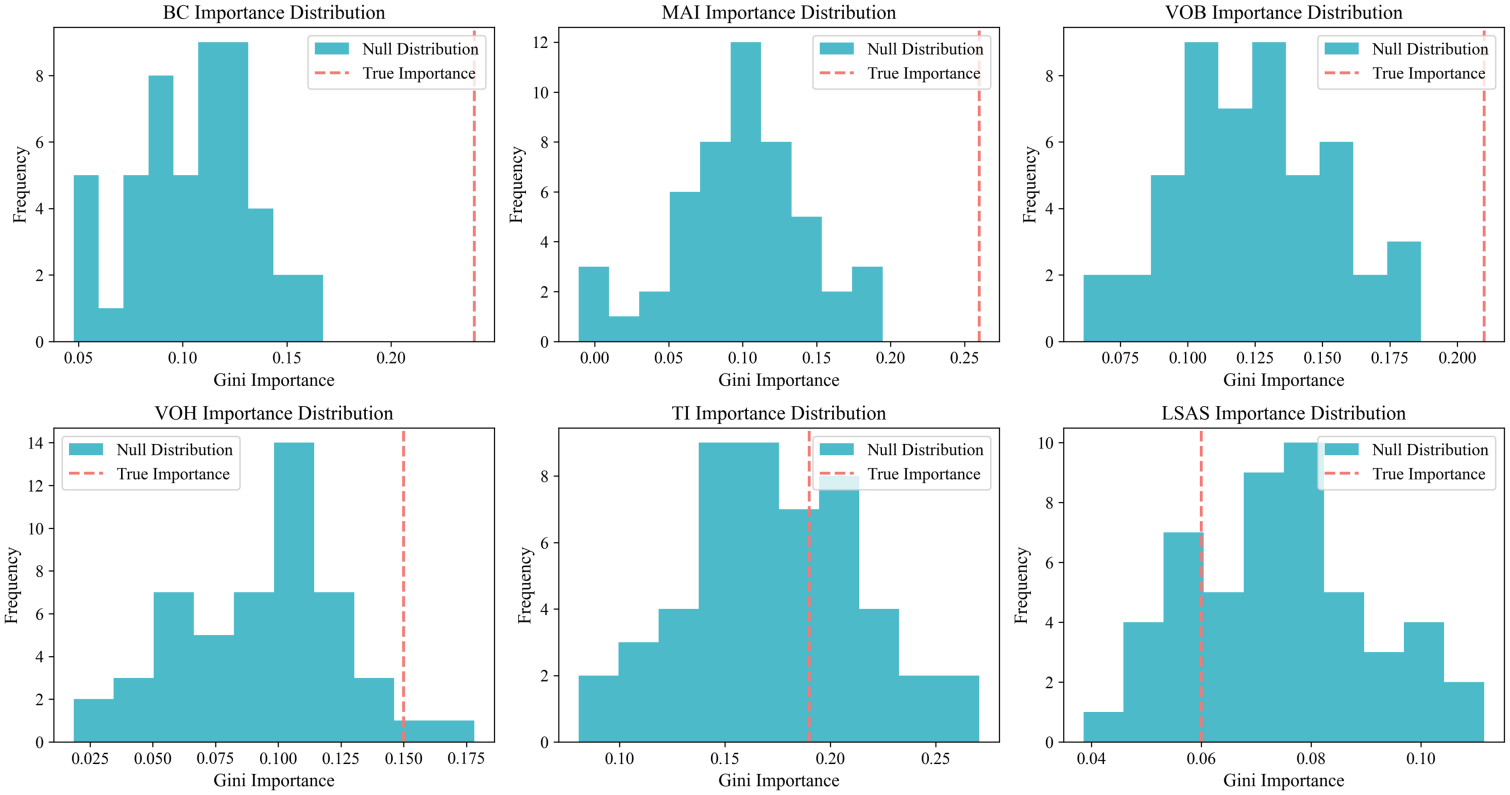
PIMP histogram. The blue bar chart in the figure illustrates the ‘null distribution’ of the importance of the corresponding feature following 50 random label permutations, while the red dotted line signifies the true Gini importance of the feature based on the original labels.

Although multi-feature fusion strategies demonstrate significant advantages in limping classification, the simple combination of all features does not necessarily enhance model performance. As shown in Table 6, the accuracy and Macro F1 scores of the full feature combination are lower than those of core feature combinations, such as BC, MAI, and VOB. This indicates that incorporating features with insufficient discriminative power may actually weaken the model’s generalization ability. Therefore, the selection of key features is critical for lameness detection. It is noteworthy that although the TI feature scored highly in the Gini importance ranking (0.19), its PIMP-corrected *p*-value was 0.314, failing to pass the significance test. Additionally, when TI was included in the model presented in Table 7, the accuracy rate actually decreased, further indicating its limited discriminative role. The results depicted in Figure 10 also show that the true Gini importance of BC, MAI, and VOB is significantly higher than that of a random distribution, indicating their key discriminative capabilities. In contrast, the true Gini importance of TI and LSAS overlaps with the null distribution, lacking independent discriminative value. These results not only reveal the differential contributions of various features to limping classification but also intuitively demonstrate the important role of PIMP correction in feature selection, providing a reliable theoretical basis for subsequent model optimization.

To validate the superiority of the proposed method for limping classification from a comprehensive perspective, this study compared the classification results of AGC-SimCC (HR-ACRNet) with those from recent related studies, as presented in Table 9. Early gait abnormality detection methods primarily relied on single features. For instance, Zin et al. (30) and Tun et al. (31) utilized limited features based on spinal height, achieving accuracy rates of only 70.3 and 81.1%, respectively. In recent years, Xin et al. (32) and Zhang et al. (33) introduced multi-feature or spatio-temporal flow fusion methods, which enhanced detection accuracy. However, the feature selection and multi-feature fusion strategy employed in this study improved the accuracy of limping classification to 91%, significantly outperforming existing methods and further validating the effectiveness of the proposed approach.

**Table 9:** Performance comparison with existing overhead view-based cow lameness detection methods.

Author (Year)	Feature(s)	Multi-feature selection	Classification	Best classifier	Accuracy (%)
Zin et al. (2022) (30)	Mean spinal height	No	Lame/Non-lame	SVM	70.30
Tun et al. (31)	Sequence of dorsal spine apex	No	Lame/Non-lame	RF	81.10
Xin et al. (32)	Spatiotemporal fused features	No	Lame/Non-lame	Cow-TSM	88.70
Zhang et al. (33)	8 motion change features	No	Sound, Mild lameness and Severe lameness	threshold discrimination method	83.05
Ours	6 motion change features	Yes	Sound, Mild lameness and Severe lameness	RF	91.00

In summary, this study proposed the AGC-SimCC (HR-ACRNet) keypoint detection method, which significantly improved the accuracy of bovine back keypoint localization, thereby establishing a foundation for subsequent feature construction. Six quantitative features for lameness under overhead conditions were designed, with MAI and VOB being the first to demonstrate strong discriminative capabilities, complementing traditional indicators such as spinal curvature and general symmetry. Furthermore, an automated lameness detection framework integrating feature selection and fusion was developed, offering advantages of non-contact operation, low cost, and ease of deployment. Overall, the proposed method provides practical solutions and technical support for intelligent health monitoring of dairy cows in livestock farms.

This study acknowledges several limitations. First, the research data were collected at a specific time from a singular indoor fixed location on one farm, which restricts the generalizability of the findings across different climatic and ground conditions. Second, critical information regarding the age, parity, and pregnancy status of the cows was not recorded during data collection, thereby limiting the potential for in-depth analysis of group differences. Finally, although the overhead view provides advantages such as reduced occlusion and ease of deployment, it also has the drawback of not allowing direct observation of the limbs and claws. The manifestations of lameness are primarily reflected in the movement characteristics of the back and head, and the relatively low video frame rate (30 fps) further complicates the capture of details related to rapid movements.

Future research will involve collecting data from dairy cows of various breeds under different production conditions across multiple time intervals to enhance the model’s generalization capability. By integrating data on disease location, etiology, and factors such as age, parity, and pregnancy status, we will perform population-specific analyses. Furthermore, we will investigate the use of high-quality data support, including higher frame rates and higher-resolution depth data, along with more refined feature construction methods, to develop a limping detection system that possesses broader applicability and population-specific characteristics. This will provide reliable technical support for automated limping detection, etiological diagnosis, and early intervention.

## 5 Conclusion

This study addresses the challenges of unclear features and low accuracy in detecting lameness in cows from an overhead view. It proposes a systematic detection method based on RGB-D data, which includes high-precision back keypoint localization, overhead view feature construction, unbiased feature selection and multi-feature fusion classification. This method effectively quantifies changes in movement and abnormal postures caused by lameness, enabling accurate classification of individuals into sound, mild lameness, and severe lameness categories. Experimental results indicate that features such as back curvature, movement asymmetry index, vertical oscillation of the back, and vertical oscillation of the head demonstrate varying degrees of lameness discrimination capability. The lameness identification accuracy achieved through multi-feature fusion reaches 91%, significantly outperforming both single-feature methods and existing overhead view detection methods. The findings validate the feasibility and advantages of using overhead view RGB-D data for automated lameness detection, providing a viable technical pathway for non-contact, low-cost lameness monitoring in dairy farms. Future research will expand the range of application scenarios and sample categories, and will explore more universally applicable overhead view lameness features to enhance the practical implementation of intelligent dairy cow health monitoring and precision livestock management.

## Data Availability

The original contributions presented in the study are included in the article/supplementary material, further inquiries can be directed to the corresponding authors.
